# Variation in assessment, diagnosis and outcome measurement in Perthes disease: a scoping review

**DOI:** 10.1177/18632521261432861

**Published:** 2026-03-24

**Authors:** Stephanie Ball, Rhianydd Thomas, Luke M. Davies, Kelly Gray, Nicole Williams, Craig F. Munns, Verity Pacey

**Affiliations:** 1 School of Health Sciences and Nursing, Macquarie University, Sydney, NSW, Australia; 2 School of Primary and Allied Health Care, Monash University, Frankston, VIC, Australia; 3 Centre for Orthopaedic and Trauma Research, University of Adelaide, Adelaide, SA, Australia; 4 Department of Orthopaedic Surgery, Women’s and Children’s Hospital, North Adelaide, SA, Australia; 5 Faculty of Medicine & Children Health Research Centre, The University of Queensland, Herston, QLD, Australia; 6 Department of Endocrinology and Diabetes, Queensland Children’s Hospital, South Brisbane, QLD, Australia

**Keywords:** Assessment, children, diagnosis, Legg–Calvé–Perthes disease, outcomes

## Abstract

**Purpose:**

Perthes disease is a rare self-limiting avascular necrosis of the developing proximal capital femoral epiphysis. Identifying methods used to assess, diagnose and determine outcomes is essential to improve consistency in research and clinical care for this patient population.

**Methods:**

The Preferred Reporting Items for Systematic Reviews and Meta-Analyses extension for scoping reviews was used. Four electronic databases (Scopus, MEDLINE, CINAHL and Embase) were searched with articles included if clinical and radiological assessments were conducted on more than 10 participants, aged 0 to <18 years with Legg–Calvé–Perthes disease post 2004.

**Results:**

From 9145 records, 32 studies were included, identifying 16 clinical assessments, 23 radiological assessments, 10 diagnostic classifications and 22 outcome measures. Overall, 64% of outcome measures identified are not currently validated for children and adolescents.

**Conclusions:**

Substantial variability in the methods used to assess, diagnose and determine outcomes in children and adolescents with Perthes disease was identified. When coupled with limited use of validated paediatric measures, this inconsistency complicates clinical decision-making, reduces consistency in patient care and prevents comparability across studies. Establishing expert consensus to determine the most appropriate, accurate and child-specific measures is needed to enhance consistency in patient care, enable more robust outcome reporting and strengthen future research in Perthes disease.

**Significance of study:**

Significant variation in diagnostic, assessment and outcome measures for Perthes disease underscores the need for validated, child-specific tools to improve clinical decision-making, facilitate comparability across studies and strengthen future research.

**Level of evidence:**

Level III, scoping review

## Introduction

Legg–Calvé–Perthes disease (Perthes disease) is a rare (0.4–29.0 per 100,000) self-limiting avascular necrosis (AVN) of the developing proximal capital femoral epiphysis, the pathophysiology of which remains unclear.^1–3^ The resulting ischaemic bone damage can progress to femoral head collapse and, in some cases, permanent hip joint deformity.^1,2^ Perthes disease predominantly affects boys (ratio 5:1), with peak incidence between 4–8 years of age.^2^ Onset after 8 years is considered late presentation and is associated with poorer long-term outcomes.^2,4–7^ The complex nature of Perthes disease has potentially devastating impacts on physical activity, participation and quality of life in children and adolescents who develop this condition both in the short and long term.^1,4,6^

Timely diagnosis is important for patients and their families to limit misdiagnosis, progression of symptoms and reduce anxiety of the unknown.^8,9^ Plain radiographs remain the primary modality for diagnostic classification, monitoring disease progression and overall outcomes.^5^ Although magnetic resonance imaging (MRI) can detect impaired vascularity at an earlier stage and delineate the extent of epiphyseal involvement, including revascularisation of femoral head, expense, accessibility and need for general anaesthesia for young children limits the routine use of MRI for serial staging of this condition.^10,11^ No international standard for diagnosing or classifying Perthes disease currently exists. Management strategies are similarly variable, with no clinical guidelines standardising treatment selection. There is increasing agreement that non-surgical management is associated with improved clinical and radiographic outcomes in less severe cases or those <6 years, given greater potential for femoral head remodelling, whereas surgical intervention is more often associated with improved outcomes in later-onset and more severe femoral head involvement.^12–14^

Inconsistent methods for assessing treatment success and disease progression in Perthes disease limit comparability across studies and impedes the development of standardised management pathways.^15^ Prior research has identified key outcome domains – developed with input from patients, families and clinicians – that encompass physical, emotional and social dimensions.^15^ Consequently, greater understanding of the assessments, diagnostic classifications and outcome measures used for this patient population is warranted to facilitate consistent treatment and research practices nationally and internationally. Therefore, the aim of this scoping review is to determine the assessments, diagnostic classifications and outcome measures used for children and adolescents with Perthes disease.

## Materials and methods

The Joanna Briggs Institute (JBI) methodology for scoping reviews^16^ and the Preferred Reporting Items for Systematic Reviews and Meta-Analyses extension for Scoping Reviews^17^ were utilised. The review protocol was registered on the Open Science Framework (OSF) database on June 23, 2024 (https://osf.io/6za3e/) as part of a broader femoral head AVN review.

Given the high number of results this review includes Perthes disease only, secondary femoral head AVN will be reported separately. Four databases (Ovid MEDLINE, Prospero, OSF and JBI Evidence Synthesis) were reviewed to ensure that no prior systematic reviews or scoping reviews were registered or currently underway.

### Eligibility criteria

Studies were included if they described diagnostic classifications, both clinical and radiological assessments, or outcome measures used in children and adolescents (aged 0–<18 years) with Perthes disease. Alternative or eponymous terminologies such as osteonecrosis, ischaemic necrosis, aseptic necrosis, femoral head necrosis or Legg–Calvé–Perthes disease were also included. Studies with a mixed adult-paediatric cohorts were included if paediatric data could be extracted separately or if the mean age of participants was below 18 years.

Studies were included if data collection and publication occurred from 2004 onwards, were peer reviewed, written in English with 10 or more participants with Perthes disease. Original experimental and non-experimental randomised and non-randomised controlled trials, observational studies both prospective and retrospective cohort, qualitative studies, case-control, and case series were included. Studies were excluded if they did not report specifically on Perthes disease, or if they were conference abstracts, opinion papers, animal studies or secondary research (systematic reviews, meta-analysis).

### Search strategy

The search was conducted on February 6, 2025. Four databases were searched including CINAHL, Embase, Ovid MEDLINE and Scopus utilising keyword, index terms and medical subject headings. The full electronic search for Medline is presented in Table 1, which was adapted for each of the included databases. Additional relevant records were sought using backward and forward chaining.

**Table 1.** Ovid Medline search strategy.

Search	Query
1	osteonecrosis/ or femur head necrosis/ or Legg-calve-Perthes disease
2	(avascular necrosis or AVN or osteonecrosis or aseptic necrosis or ischaemic necrosis or ischemic necrosis or osteochondrosis or articular osteochondrosis or ‘avascular necrosis of femoral head’ or Legg Calve or Perthes or Perthes disease).ti,ab.
3	1 or 2
4	lower extremity/ or hip/
5	(lower extremity or lower limb* or hip* or femoral head* or femur*).ti,ab.
6	4 or 5
7	adolescent/ or child/ or child, preschool/
8	(child or children or adolesc* or paediatric* or pediatric* or schoolchild* or boy or boys or girl* or pubescen* or juvenile* or teen* or youth* or pre-pubes* or prepubesc*).ti,ab.
9	7 or 8
10	3 and 6 and 9
11	diagnosis/ or clinical decision-making/ or delayed diagnosis/ or diagnosis, differential/ or ‘diagnostic techniques and procedures’/ or early diagnosis/
12	(diagnos*or diagnostic criteria or classific*).ti,ab.
13	11 or 12
14	Outcome assessment, Health Care/ or Patient Reported Outcome Measures/ or Patient Outcome Assessment/ or Treatment Outcome/
15	(outcome* or outcome measures or evaluat*).ti,ab.
16	14 or 15
17	Pain Perception/ or Musculoskeletal Pain/ or Pain Measurement/ or Chronic Pain/ or Pain
18	(pain or chronic pain or musculoskeletal pain or pain perception or pain measurement).ti,ab.
19	‘Quality of Life’/
20	(quality of life or physical activit* or participat*).ti,ab.
21	gait/ or gait analysis/ or muscle strength/ or ‘range of motion, articular’/
22	(gait or gait analys* or mobil* or walk* or strength or muscle strength or range of motion or range of movement or ROM).ti,ab.
23	17 or 18 or 19 or 20 or 21 or 22
24	10 and (13 or 16 or 23)
25	Limit 25 to English language

### Study selection

Studies identified from the search were uploaded into Endnote (End Note 20, Clarivate Analytics, Philadelphia, PA, USA), transferred into Covidence (Veritas Health Innovations, Melbourne, Australia), with duplicates removed. Two reviewers (S.B., R.T.) screened titles, abstracts, and assessed potentially relevant full text records independently. Resolution of any disagreements between reviewers was resolved through discussion, and where required, with an additional reviewer (V.P., N.W.).

### Data extraction

Data were extracted from all eligible studies by one reviewer (S.B.), with 20% of data checked and agreed upon by a second reviewer (K.G., R.T.). A purpose-built spreadsheet in Microsoft Excel was created to extract the following: author, year, country, study design, participant characteristics, laterality, surgical procedures and adverse events, timing of follow-up, health professional involvement, details of any assessments, diagnostic classifications or outcome measures used.

### Data analysis

Quantitative data were descriptively summarised using frequencies and percentages. Content analysis was performed to report on the assessments, diagnostic classifications and outcome measures used. Assessments and outcome measures were mapped to the international classifications of functioning and the domains of the core outcome set for Perthes disease.^15,18^ A literature search was conducted to examine the psychometric properties and development of outcome measures.

## Results

**Figure 1.**
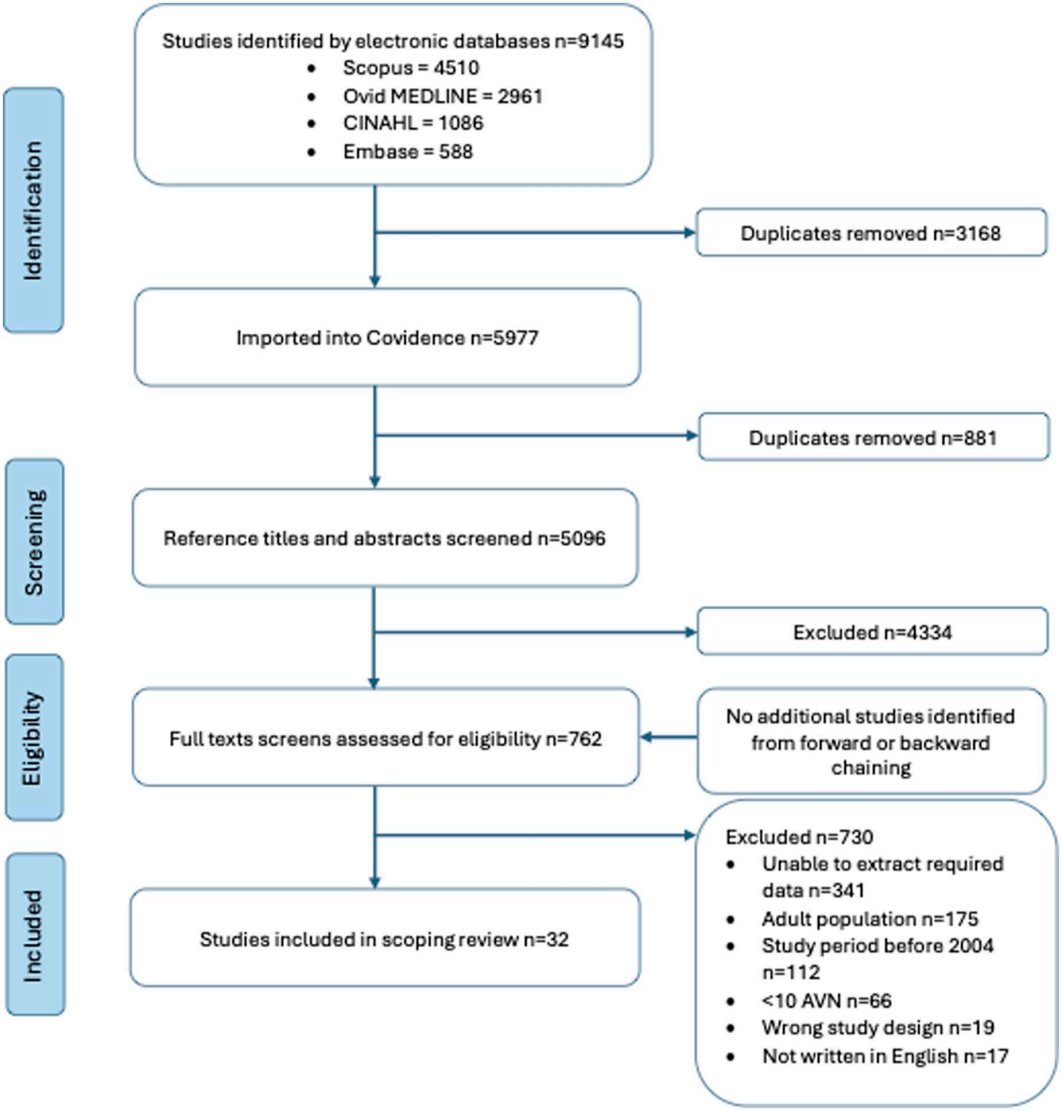
PRISMA flow diagram of inclusion process for studies. n: Number; AVN: Avascular necrosis; PRISMA: Preferred Reporting Items for Systematic Reviews and Meta-Analyses.

A total of 9145 records were retrieved; after duplicate removal, 5096 titles and abstracts were screened. Full text review of 762 articles resulted in 32 studies meeting inclusion criteria (Figure 1). Data were collected and published between 2004 and 2024, with 16 countries contributing to the data. Just under half (47%) of studies reported health professional involvement, with paediatric orthopaedic surgeons or orthopaedic surgeons (paediatric not specified) the most prevalent (*n* = 14 studies, 43%).

### Study characteristics

Study characteristics can be seen in Online Supplemental 1. Thirty-two studies included 2149 children and adolescents. The majority were males (male:female = 3.6:1), with a median age of 8.4 years (range 4.8–12.7 years). Laterality was reported in 27 studies; the left hip marginally more affected than the right (left:right = 493:471). Six (18%) studies used a single assessment time point. Thirteen of the 14 core outcome domains were examined by included studies, no study included all domains, and none assessed sleep quality (Online Supplemental 2). Twenty-six (82%) studies assessed pre- and post-surgical intervention, with follow-up periods of 0.75–8.7 years (median 3.1 years), with half reporting on adverse surgical events. One (3%) study investigated aetiological and prevalence factors.

### Assessments

**Figure 2.**
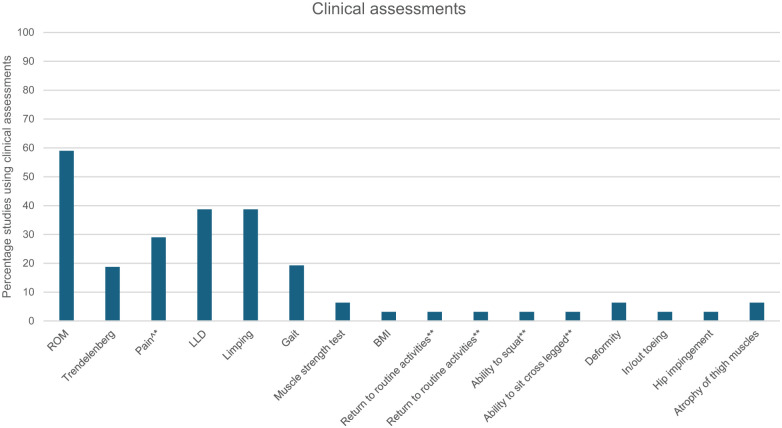
Clinical assessments used by studies. ROM: Range of motion; LLD: Leg length discrepancy; BMI: Body mass index. ^*Scale not specified. **Assessing at activities and participation level on World Health Organisation, International Classification of Functioning, Disability and Health.

**Figure 3.**
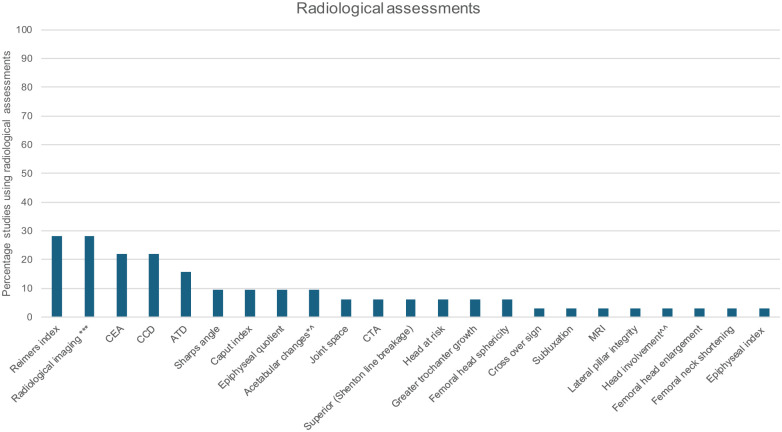
Radiological assessments used by studies. CEA: Centre edge angle (of Wiberg); CCD: Caput-collum-diaphyseal angle; ATD: Articulotrochanteric distance; MRI: Magnetic resonance imaging. ***No specification of measures used. *^Height and width; CTA: Centre-trochanteric angle. ^^Percentage.

Sixteen clinical (Figure 2) and 23 radiological assessments (Figure 3) were reported in 25 (78%) studies. Multiple assessments were frequently used concurrently, with a median of 6 (range 1–11) per study. Clinical and radiological assessments were used in 20 (62%) studies, while 4 (16%) used only clinical, and 1 (4%) used only radiological assessments. In all studies where surgical procedures were conducted (*n* = 26 studies, 81%), radiological outcome measures or assessments were used pre- and post- operatively. All studies evaluated at the body structure and function level, while evaluation of activity and participation occurred in three (12%) studies. In all studies, methods of assessments were conducted were either not described in detail or description was insufficient. For example, hip range of movement (ROM) was performed in 18 (72%) studies, 16 (89%) did not specify measurement procedures or planes of movement, and 1 (5%) study specified ROM was assessed passively.

### Diagnostic classifications

**Figure 4.**
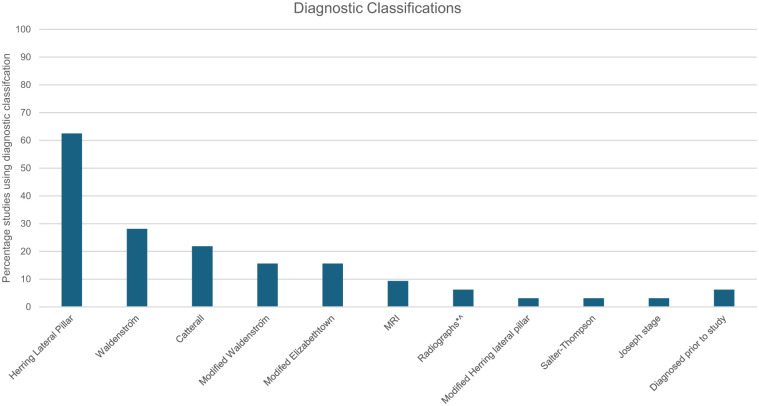
Diagnostic classifications used by studies. Herring Lateral Pillar Classification; Waldenström Classification; Catterall Classification; Modified Waldenström Classification; Modified Elizabethtown Classification; MRI: Magnetic resonance imaging. ^*no other details specified; Modified Herring Lateral Pillar Classification; Salter-Thompson Classification.

Diagnostic classifications were reported in 30 (94%) studies (Figure 4). All studies utilised radiographs for diagnosis, with three (9%) studies also using MRI. Ten different classification tools were identified; Herring Lateral Pillar classification was most frequently used (*n* = 20 studies, 63%), followed by Waldenström (*n* = 8 studies, 25%) and Catterall (*n* = 7 studies, 22%) classifications. Over half the studies used more than 1 classification to diagnose: 11 (34%) used a single classification, 14 (44%) used 2, 3 (9%) used 3, and 1 (3%) used 4 distinct classifications. Classifications provided measures of severity and/or prognostic information. Prognostic tools were utilised in 21 (72%) studies, while diagnostic tools used in 19 (66%) studies; 10 (31%) studies utilised both classification types.

### Outcome measures

**Figure 5.**
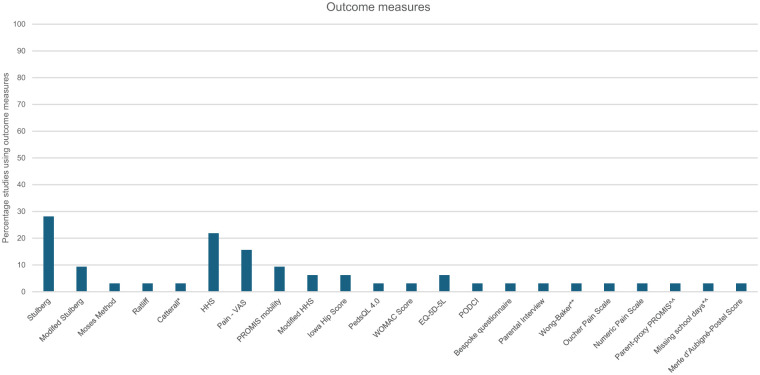
Outcome measures used by studies. HHS: Harris Hip Score; VAS: Visual analogue scale; PROMIS Mobility: Patient Reported Outcome Measurement Information System – Mobility; PedsQL4.0: Paediatric Quality of Life Inventory; WOMAC: Western Ontario and McMaster Universities Osteoarthritis Index; EQ-5D-5L: EquroQual-5 Dimension 5 questions; PODCI: Paediatric Outcomes Data Collection Instrument; Stulberg classification; Modified Stulberg classification; Ratliff: Ratliff’s classification of avascular necrosis. *Catterall post-operative classification. **Wong Baker FACES Pain Rating Scale. Parent Proxy PROMIS^^: The National Institutes of Health’s Patient- Reported Outcome Measurement Information System (PROMIS) – parent reported. *^Due to pain.

Outcome measures were reported in 26 (81%) studies, using 22 different outcome measures (17 clinical, 5 radiological) (Figure 5, Online Supplementals 1 and 2). Over half of these measures (63%) were used once in a single study. The Stulberg and Modified Stulberg Classifications were the most frequently used radiological measures to evaluate post-operative outcomes (*n* = 12 studies, 37%). The Harris Hip Score (*n* = 7 studies, 22%) and Patient Reported Outcome Measurement Information System mobility (*n* = 3 studies, 9%) were the most frequently used clinical outcome measures, addressing the domains of pain, activities of daily living (including gait) and hip mobility. Health-related quality of life was assessed using five outcome measures in eight (25%) studies. Under half (36%) of the outcome measures are considered validated and reliable for children and adolescents. Thirteen are currently not psychometrically tested for use with this patient population, while six of these were originally developed for children/adolescents (Online Supplemental 2).

## Discussion

To our knowledge, this is the first scoping review to explore the specific assessments, diagnostic classifications and outcome measures used in children and adolescents with Perthes disease. The findings demonstrate substantial variability across diagnostic and evaluative methods. Building consensus by experts on the most relevant, specific and reliable assessment measures and diagnostic classifications would improve outcomes and foster evidence-based healthcare.

Radiological imaging was the predominant method of diagnosing and monitoring outcomes for all participants, with less frequent use of MRI. While this reflects consistency in imaging practices, considerable variation in classifications applied to interpret the imaging was evident. All methods have inherent limitations, the most notable being classifications lack validity until fragmentation has occurred,^19–21^ limiting early intervention options and necessitating a ‘watch and classify’ approach. This is particularly problematic for older children, who possess a more limited femoral head remodelling period and may experience prolonged diagnostic uncertainty and unpredictable clinical outcomes. Radiographs are accessible and easy to administer, whereas MRI enables earlier detection of perfusion changes to the femoral epiphysis, but may not be suitable for ongoing monitoring.^11,22^ Interpretation reliability is associated with clinician experience, and among classification systems, Lateral Pillar classification demonstrates higher interobserver reliability compared to Catterall and Salter-Thompson classifications.^23,24^ Robust reliability studies including MRI and radiological imaging is warranted to establish the most relevant, specific and reliable diagnostic classifications to improve consistency of early diagnosis, support data sharing and enhance evidence-based healthcare.

The paucity of validated hip outcomes for children and adolescents requires attention to ensure that measurements are meaningful to the patients and psychometrically sound. Tools developed for adults, often targeting arthritic hips, suffer from ceiling effects and determine outcomes that may not be relevant or appropriate to children and adolescents.^25,26^ Patient-derived outcomes are essential for capturing meaningful health outcomes, detecting minimal important change and informing patient-centred care.^25^ While evaluating the confidence of treatment effects was beyond this review’s scope, the limited use of validated and reliable outcome measures in the included studies may potentially bias study conclusions. A qualitative study involving children with Perthes disease, their families and clinicians identified widespread variation in routine treatment practices, largely attributed to the absence of appropriate outcome measures tailored to this patient population.^27^ In the absence of robust clinical trials guiding clinicians and researchers, obtaining consensus among experts should be considered to facilitate a standardised approach to assessment and evaluation of outcomes in the interim.

While half of the identified outcome domains for Perthes disease were captured by most studies, the methods used to assess these outcomes varied considerably. The existing core outcome set, while developed for research purposes, provides a fundamental guide for clinical practice, while not specifying the methods to measure each domain.^15^ The absence of methodological guidance has contributed to heterogeneous assessment practices and inconsistent approaches across studies. Furthermore, most studies provided limited or no detail on the assessment methodology, leaving the specific measurements or procedures unclear. Collectively, these issues underscore the need for methodological standardisation to improve the rigour of outcome measurement, support meaningful comparisons across studies, facilitate future meta-analyses and strengthen the evidence base required to inform consistent and effective clinical care.^28^

A greater understanding of the impact on daily function, lower limb pain and quality of life upon children and their families during management, and what is considered successful, is necessary to progress clinical practice and research. Clinicians identified their main goals of minimising lower limb pain and increasing patient function when managing children with Perthes disease.^27^ While the domain of activity participation and subjective measures of pain and quality of life were investigated by some studies, greater emphasis on these aspects is warranted in future research. Importantly, children/adolescents and their families have expressed their desire to be involved in the decision-making process.^27^ Incorporating the perspectives of key stakeholders – particularly patients and their families – into the development of appropriate self-reported and objective outcome measures may enhance research and clinical practice.

While this study was limited by including studies written only in English, with 10 or more participants with Perthes disease, it focused on both clinical and radiological outcomes for children and adolescents in this population. Some assessments, diagnostic classifications and outcome measures may have been missed because of the inclusion and exclusion criteria, most notably the requirement of both clinical and radiological assessments. However, a large number of studies were included in initial screening.

The results of this study identified substantial variability in the assessments, diagnostic classifications and outcome measures used to evaluate and monitor children and adolescents with Perthes disease. Limited validated outcome measures were evident for this patient cohort. Such heterogeneity complicates clinical decision-making, reduces consistency in patient care and limits the capacity to compare outcomes across studies. Future studies should prioritise establishing expert consensus to determine the most appropriate, accurate and child-specific measures, alongside the development of clinical practice guidelines, to support more consistent diagnosis, monitoring and prognostic evaluation. Additionally, the validation of paediatric-specific outcome measures relevant to Perthes disease, while considering the impact of the condition and its treatments on children and their families, will be important for improving evidence-informed care and strengthening future research in this population.

## Supplemental Material

sj-docx-4-cho-10.1177_18632521261432861 – Supplemental material for Variation in assessment, diagnosis and outcome measurement in Perthes disease: a scoping review Supplemental material, sj-docx-4-cho-10.1177_18632521261432861 for Variation in assessment, diagnosis and outcome measurement in Perthes disease: a scoping review by Stephanie Ball, Rhianydd Thomas, Luke M. Davies, Kelly Gray, Nicole Williams, Craig F. Munns and Verity Pacey in Journal of Children's Orthopaedics

sj-pdf-1-cho-10.1177_18632521261432861 – Supplemental material for Variation in assessment, diagnosis and outcome measurement in Perthes disease: a scoping review Supplemental material, sj-pdf-1-cho-10.1177_18632521261432861 for Variation in assessment, diagnosis and outcome measurement in Perthes disease: a scoping review by Stephanie Ball, Rhianydd Thomas, Luke M. Davies, Kelly Gray, Nicole Williams, Craig F. Munns and Verity Pacey in Journal of Children's Orthopaedics

sj-pdf-2-cho-10.1177_18632521261432861 – Supplemental material for Variation in assessment, diagnosis and outcome measurement in Perthes disease: a scoping review Supplemental material, sj-pdf-2-cho-10.1177_18632521261432861 for Variation in assessment, diagnosis and outcome measurement in Perthes disease: a scoping review by Stephanie Ball, Rhianydd Thomas, Luke M. Davies, Kelly Gray, Nicole Williams, Craig F. Munns and Verity Pacey in Journal of Children's Orthopaedics

sj-pdf-3-cho-10.1177_18632521261432861 – Supplemental material for Variation in assessment, diagnosis and outcome measurement in Perthes disease: a scoping review Supplemental material, sj-pdf-3-cho-10.1177_18632521261432861 for Variation in assessment, diagnosis and outcome measurement in Perthes disease: a scoping review by Stephanie Ball, Rhianydd Thomas, Luke M. Davies, Kelly Gray, Nicole Williams, Craig F. Munns and Verity Pacey in Journal of Children's Orthopaedics
