# Supplementary material for: Variation in assessment, diagnosis and outcome measurement in Perthes disease: a scoping review
Source: J Child Orthop. 2026 Mar 24;20(3):247–55. doi: 10.1177/18632521261432861 (PMC13017626; doi:10.1177/18632521261432861)
Supplement: sj-pdf-2-cho-10.1177_18632521261432861 – Supplemental material for Variation in assessment, diagnosis and outcome measurement in Perthes disease: a scoping review [file sj-pdf-2-cho-10.1177_18632521261432861.pdf]

Supplemental Table 1. Study characteristics, assessments, diagnostic classification and outcome measures for all studies

Author, year, study type, country	Study period & participant characteristics	Diagnosis & health professional involved	Assessments	Diagnostic Classifications	Outcome Measures
**Perthes disease only studies**					
Amer 2011 [1]<br><br>Prospective, single centre<br><br>Egypt	2004-2008<br><br>n=30<br><br>n with AVN=30<br><br>M=20, F=10<br><br>Diagnosis age >8yr<br><br>Mean age at surgery 9.7yr<br><br>Follow up 3.6yr	Perthes disease, late onset (>8yr)<br><br>Health professionals involved: does not specify	Hip ROM<br><br>Trendelenburg gait<br><br>Radiological imaging:<br>Superior & Lateral subluxation	The Herring Lateral Pillar Classification<br><br>Waldenström Classification of Perthes Disease	*Clinical*<br><br>Pain - VAS

Moghadam 2013 [2]<br><br>Prospective, single centre<br><br>Iran	2008-2013<br><br>n=29<br><br>n with AVN=29<br><br>M=25, F=4<br><br>Symptoms present mean 7mon prior to surgery<br><br>Mean age at study 9yr<br><br>Follow up, not specified	Perthes disease<br><br>Health professionals involved: does not specify	Limb length discrepancy<br><br>Hip ROM<br><br>Limping<br><br>Trendelenburg test	The Herring Lateral Pillar Classification	*Radiological*<br><br>AP and frog leg radiographs; Flattening of femoral head, Acetabular changes, Femoral neck shortening, Trochanter enlargement, Abnormal femoral neck angel and Femoral head enlargement.<br><br>Osteoarthritis signs<br><br>*Clinical*<br><br>Pain-VAS
Yavuz 2014 [3]	2004-2009<br><br>n=17 (18 hips)	Perthes disease	Limp	The Herring Lateral Pillar Classification	*Radiological*<br><br>Modified Stulberg Classification

Retrospective, single centre<br><br>Turkey	n with AVN=17 (18 hips)<br><br>M=16, F=1<br><br>Age at diagnosis and at study mean 8.8yr<br><br>Follow up 6.5yr	Health professionals involved: not specified	Limb length discrepancy<br><br>Hip ROM<br><br>Pain with activity<br><br>Radiological imaging:<br>CEA<br><br>Sharp acetabular index<br><br>Acetabular depth index<br><br>Acetabular covering percentage<br><br>Cross-over sign		
Camurcu 2015 [4]	2007-2011<br><br>n=43 (50 hips)	Perthes disease	Hip ROM<br><br>Pain	Waldenström Classification of Perthes Disease	*Radiological*<br><br>Stulberg Classification

Retrospective, single centre<br><br>Turkey	n with AVN=43<br><br>M=34, F=9<br><br>Age at diagnosis not specified, 63mon from first admission to surgery<br><br>Mean age at surgery 9.4yr<br><br>Follow up 2.8yr	Health professionals involved: does not specify	Gait<br><br>Leg length discrepancy<br><br>Trendelenburg sign<br><br>FABER and FADIR<br><br>Radiological imaging:<br>AP and von Rosen<br><br>CEA	Catterall Classification<br><br>The Herring Lateral Pillar Classification	*Clinical*<br><br>Harris Hip Score
Elzohairy 2016 [5]<br><br>Retrospective, single centre<br><br>Egypt	2005-2011<br><br>n=23<br><br>n with AVN=23<br><br>M=23, F=0	Perthes disease<br><br>Health professionals involved: does not specify	Hip pain<br><br>Limping<br><br>Knee pain	Catterall Classification<br><br>The Herring Lateral Pillar Classification	*Clinical*<br><br>Iowa Clinical Hip Score

	Age at diagnosis not specified<br><br>Mean age 7.8yr at surgery<br><br>Follow up mean 3yr		Leg length discrepancy<br><br>Hip ROM<br><br>Gluteal weakness<br><br>Radiological imaging:<br>AP x-rays in neutral, abduction, and abduction–internal, frog-leg lateral views:<br><br>Extrusion index<br><br>CEA<br><br>Neck shaft angel		
Bhuyan 2016 [6]<br><br>Retrospective	2005-2012<br><br>n=23	Perthes disease	Presenting symptoms: pain, limp, or both	Waldenström Classification of Perthes Disease	*Radiological*<br><br>Stulberg Classification

India	n with AVN=23<br><br>M=19, F=4<br><br>Onset of symptoms mean 6.1yr, diagnosis mean 3mon later<br><br>Mean age at surgery 6.6yr<br><br>Follow up 5.4yr	Health professionals involved: does not specify	Hip ROM<br><br>Radiological imaging:<br>Epiphyseal extrusion index<br><br>MRI<br><br>“head-at-risk” signs: The radiological signs of head-at-risk consisting of Gage sign, calcification lateral to the epiphysis, lateral subluxation, diffuse metaphyseal reaction and horizontal growth plate were examined in all patients.	Joseph stage<br><br>Catterall Classification<br><br>The Herring Lateral Pillar Classification	Mose’s method (femoral head sphericity)<br><br>*Clinical and radiological*<br><br>Ratliff Classification
Aguado-Maestro 2016 [7]	2010-2014<br><br>n=13 (13 hips)	Perthes disease – late onset	Hip joint balance is studied before and after the operation (passive joint	Waldenström Classification of Perthes Disease	*Radiological*<br><br>Modified Stulberg

Ambispective, single centre<br><br>Spain	n with AVN=13<br><br>M=9, F=4<br><br>Age at diagnosis mean10.32yr<br><br>Mean age at surgery 11.93yr<br><br>Follow up 1.75yr	Health professionals involved: does not specify	balance, flexion-abduction, internal rotation, external rotation)<br><br>Gluteal insufficiency (Trendelenburg sign – yes/no)<br><br>Limp (yes/no)<br><br>Radiological imaging:<br>Signs of risk: lateral calcification, metaphysis rarefication, Gage's sign, subluxations, subluxation with hinge deformity		(due to skeletal immaturity in some patients)<br><br>Sphericity of femoral compared to preoperative<br><br>*Clinical*<br>Pain – VAS (Before surgery, during treatment with fixator insitu and after treatment when fixator not insitu are compared)
Keun-Sang 2017 [8]	2006-2013<br><br>n=19	Perthes disease	Radiological imaging: AP	The Herring Lateral Pillar Classification	*Radiological*<br><br>Modified Stulberg Classification

Retrospective, single centre<br><br>Korea	n with AVN=19<br><br>M=19, F=0<br><br>Age at diagnosis not specified<br><br>Mean age for study not specified<br><br>Follow up 8yr	Group 1: <8yr, mean age 6.4yr<br><br>Group 2: >8yr, mean age 8.5yr<br><br>Health professionals involved: three orthopaedic surgeons independently assessed the radiographic measurements. All measurements were collected by research assistant who otherwise did not participate in the study.	Growth of the greater trochanter<br><br>Articulo-trochanteric distance<br><br>Center-trochanteric distance<br><br>Neck shaft angle		*Clinical*<br><br>Iowa hip score

Joshi 2018 [9]<br><br>Prospective cohort, single centre<br><br>India	2008-2014<br><br>n=15<br><br>n with AVN=15<br><br>M=13, F=2<br><br>Mean age at presentation and study 9.4yr<br><br>Follow up mean 3.4yr	Perthes disease<br><br>Health professionals involved: all operated on by senior orthopaedic surgeon.	Hip ROM<br><br>Deformity<br><br>Gait<br><br>Pain<br><br>Limb length discrepancy (cm)<br><br>Ability to sit cross legged<br><br>Ability to squat<br><br>Radiological imaging:<br>AP and lateral:<br><br>Caput index<br><br>Epiphyseal quotient	Modified Elizabethtown Classification<br><br>MRI: to rule out revascularization stage.	*Clinical*<br><br>Harris Hips Score

			Articulo-trochanteric distance		
Kanatli 2019 [10]<br><br>Retrospective, single centre<br><br>Turkey	2010-2016<br><br>n=10<br><br>n with AVN=10<br><br>Sex not specified<br><br>Mean time of symptoms prior to surgery 27mon<br><br>Mean age at surgery 12.7yr<br><br>Mean follow up 4.6yr	Perthes disease<br><br>Health professionals involved: all operations completed by the same senior orthopaedic surgeon		Waldenström Classification of Perthes Disease	*Radiological*<br><br>Stulberg Classification<br><br>*Clinical*<br><br>Modified Harris Hip Score

Leo 2019 [11]<br><br>Concurrent triangulation mixed methods study, single centre<br><br>United Kingdom	2007-2017<br><br>n=12<br><br>n with AVN =12<br><br>M=10, F=2<br><br>Age at diagnosis not specified<br><br>Mean age at study 7.1yr<br><br>Follow up: one-time assessment	Perthes disease<br><br>Children with Perthes disease n=12<br><br>Parents/guardians of children with Perthes disease n=18<br><br>Health professionals involved: Both the children's questionnaire and the parents' interview were designed, conducted, and analysed by members of the team with a background/training in		Diagnosed prior to study, details not provided	*Clinical*<br><br>Children completed a bespoke questionnaire created by the. Questions related to social, physical, and emotional impact of Perthes disease (such as pain, the impact of the disease on social relationships, and the influences of it on daily life activities).<br><br>Parents engaged in a semi structured interview. The interviews aimed to explore participants' experience of the disease and the impact of Perthes disease on their lives.

		psychology/qualitative research			
Matsumoto 2020 [12]<br><br>International multicentre validity study<br><br>United States of America & India	2013-2018<br><br>n=190<br><br>n with AVN=190<br><br>M=154, F=36<br><br>Age at diagnosis not specified<br><br>Mean age at study 10.4yr<br><br>Follow up: one-time assessment	Perthes disease<br><br>Health professionals involved: does not specify		Modified Waldenström Classification of Perthes Disease	*Clinical*<br><br>PROMIS<br><br>The following 3 domains and 7 subdomains were selected:<br>(1) Physical Health: Physical Function-Mobility, Pain- Interference, and Fatigue;<br><br>(2) Mental Health: Emotional Distress-Depressive Symptoms, Emotional Distress- Anxiety, and Emotional Distress-Anger;<br><br>(3) Social Health: Peer Relationships.

Haskel 2020 [13]<br><br>Retrospective comparative study<br><br>United States of America	2006-2010<br><br>n=43<br><br>n with AVN=43<br><br>M=40, F=3<br><br>Age at diagnosis not specified<br><br>Median age at surgery 8yr<br><br>Follow up median 6.6yr	Perthes disease<br><br>Group A: greater trochanteric descent at median 7.7yr, n=12<br><br>Group B: greater trochanteric epiphysiodesis median 8.3yr, n=11<br><br>Group C: propensity matched cohort – controls, no surgery, median 8yr, n=20<br><br>Health professionals involved: Orthopaedic surgeon<br>Radiographs assessed by 2 experienced	Observational gait analysis	Waldenström Classification of Perthes Disease	*Radiological*<br><br>Stulberg Classification<br><br>Neck-shaft angle<br><br>*Clinical*<br><br>Abductor strength: muscle strength score on a scale of 0 to 10 points, per a modification of the Medical Research Council scale

		orthopaedic surgeons who were independent from treatment.<br>Muscle strength testing assessed by 2 independent paediatric orthopaedic fellow and physical therapists			
El-Harbiti 2020 [14]<br><br>Cohort, multicentre<br><br>Sweden	2015-2019<br><br>n=192 (199 hips)<br><br>n with AVN=192<br><br>M=156, F=43	Perthes disease<br><br>Health professionals involved: does not specify	Hip ROM<br><br>Trendelenburg's sign<br><br>Leg length discrepancy<br><br>Radiological imaging:	The Herring Lateral Pillar Classification	

	Mean age diagnosis and in study 5.9yr<br><br>Follow up 2yr		Articulo-trochanteric distance<br><br>Reimer's index		
Laine 2021 [15]<br><br>Retrospective, multicentre<br><br>China, India, Korea, Canada, United States of America, Germany	2012-2018<br><br>n=209 (218 hips)<br><br>n with AVN=209<br><br>M=170, F=28<br><br>Age at diagnosis & study mean 7.9yr<br><br>Follow up: one-time assessment	Perthes disease<br><br>Health professionals involved: Limp assessment by the 'treating provider'	Limp severity (no, moderate, severe)<br><br>BMI	Modified Waldenström Classification of Perthes disease	*Clinical*<br><br>Pain: 11-point scale ranging from 0 (no pain) and 10 (worst pain imaginable)<br><br>Missing days from school secondary to pain

Dang-Huy 2021 [16]<br><br>Retrospective, single centre<br><br>United States of America	2013-2020<br><br>n=100<br><br>n with AVN=100<br><br>M=85, F=15<br><br>Age at diagnosis median 8yr<br><br>Median age 9yr in study<br><br>Follow up: one-time assessment	Perthes disease<br><br>Health professionals involved: Orthopaedic surgeon assigned modified Waldenström Classification and Herring Lateral Pillar Classification		Modified Waldenström Classification of Perthes Disease<br><br>The Herring Lateral Pillar Classification	*Clinical*<br><br>PROMIS:<br>Aspects of the questionnaire; Mobility, pain interference, fatigue, depressive symptoms, anxiety, peer relationships.
Do 2022 [17]<br><br>Retrospective cross	2016-2021<br><br>n=92<br><br>n with AVN=92	Perthes disease<br><br>Group A: No restriction to WB n=21		Modified Waldenström Classification of Perthes Disease	*Clinical*<br><br>PROMIS;<br>The parents or guardians of patients completed PROMIS.

sectional, single centre<br><br>United States of America	M=79, F=13<br><br>Age at diagnosis median 5yr<br><br>Median age 6yr at study<br><br>Follow up: one-time assessment	Group B: Mild restriction to WB n=21<br><br>Moderate restriction to WB n=28<br><br>Severe restriction to WB n=22<br><br>Health professionals involved: Orthopaedic surgeon assigned modified Waldenström stages & Lateral Pillar Classification		The Herring Lateral Pillar Classification	Activity restriction status
Ghaznavi 2022 [18]<br><br>Retrospective	2015-2018<br><br>n=21	Perthes disease<br><br>Health professionals involved: radiologist	Hip ROM<br><br>Deformity	Modified Elizabethtown Classification	

Iran	n with AVN=21<br><br>M=17, F=4<br><br>Age at initial symptoms 7.7mo prior to surgery<br><br>Mean age at surgery 8.7yr<br><br>Follow up 2yr	and paediatric orthopaedic fellow	Gait<br><br>Limb-length discrepancy<br><br>Pain<br><br>Radiological imaging:<br>AP & lateral x-ray;<br>Caput index<br>Epiphyseal quotient	Modified Lateral Pillar Classification	
Perry 2022 [19]<br><br>Prospective cohort, multicentre	2016-2017<br><br>n=371 (396 hips)<br><br>n with AVN=371<br><br>M=228, F=83	Perthes disease<br><br>Health professionals involved:<br>Orthopaedic surgeon, clinician who make	Hip stiffness defined as ‘significantly limited abduction’ in the clinic environment: none/minimal, or stiff hip.	Modified Waldenström Classification of Perthes disease<br><br>Radiological diagnosis of Perthes disease, defined using the	*Radiological*<br><br>Stulberg Classification<br><br>*Clinical*<br><br>PedsQL 4.0

United Kingdom	Age at diagnosis not specified<br><br>Median age at study 5.3yr<br><br>Follow up 2yr	treating decisions, surgical trainees	Radiological imaging:<br><br>Lateral pillar integrity<br><br>Femoral head involvement (%)	typical radiological features within the femoral epiphysis: flattening, sclerosis, fragmentation, collapse, and reossification. Features could be evident on either plain radiographs or MRI.	EQ-5D- y<br><br>Wong-Baker faces pain rating scale.
Al-Osaimi 2022 [20]<br><br>Retrospective cross sectional, single centre<br><br>Saudi Arabia	2005-2015<br><br>n=23 (27 hips)<br><br>n with AVN=23<br><br>m=20, F=3<br><br>Age at diagnosis not specified	Perthes disease<br><br>Health professionals involved: authors collected the retrospective data from medical records	Hip pain (patient reported, pre and post treatment)<br><br>Leg length discrepancy<br><br>Limping<br><br>In/out toeing	The Herring Lateral Pillar Classification	

	Mean age 7.1yr in study<br><br>Follow up, does not specify		Knee pain<br><br>Hip ROM<br><br>Radiological improvement		
Arsalan 2023 [21]<br><br>Cross-sectional, single centre<br><br>Pakistan	2021-2022<br><br>n=59 (61 hips)<br><br>n with AVN=59<br><br>M=39, F=29<br><br>Diagnosis age not specified.<br><br>Mean age during study 4.76yr	Perthes disease<br><br>Health professionals involved: does not specify	Pain (hip or groyne)<br><br>Limping<br><br>Nighttime pain<br><br>Morning stiffness/ Joint stiffness<br><br>Restricted hip movement<br><br>Atrophy of thigh muscles	Diagnosed prior to study, not specified	*Clinical*<br><br>Harris Hip Score

	Follow up, does not specify		Trendelenburg gait<br><br>Leg length discrepancy		
Eidelman 2023 [22]<br><br>Retrospective, single centre<br><br>Israel	2013-2018<br><br>n=15<br><br>n with AVN=15<br><br>M=8, F=7<br><br>Age at diagnosis 1mon prior to surgery<br><br>Mean age 7.5yr at surgery<br><br>Follow up mean 3.2yr	Perthes disease<br><br>Health professionals involved: does not specify	Limp; yes/no<br><br>Hip ROM<br><br>Radiological imaging:<br>Epiphyseal-trochanteric distance	Waldenström Classification of Perthes Disease<br><br>Salter-Thompson Classification<br><br>Herring Lateral Pillar Classification	*Radiological*<br><br>Stulberg Classification<br><br>*Clinical*<br><br>Harris Hip Score<br><br>WOMAC score

Gumin Tayeng 2023 [23]<br><br>Prospective cohort, single centre<br><br>India	2018-2018<br><br>n=25<br><br>n with AVN=25<br><br>M=18, F=7<br><br>Age at diagnosis not specified<br><br>Mean age at study 8.2yr<br><br>Follow up 9mon	Perthes disease<br><br>Health professionals involved: does not specify	Limb length discrepancy (mm)<br><br>Pain<br><br>Stiffness<br><br>Hip ROM<br><br>Muscle wasting (mm)<br><br>Limping (yes/no)<br><br>Radiological imaging:<br>AP & frog-leg lateral views of the hip:<br>Neck-shaft angle	The Herring Lateral Pillar Classification<br><br>Catterall Classification	*Radiological*<br><br>Catterall's postoperative Classification<br><br>*Clinical*<br><br>Harris Hip Score

Lal 2023 [24]<br><br>Quasi-prospective observational analysis, single centre<br><br>India	2016-2021<br><br>n=13<br><br>n with AVN=13<br><br>M=7, F=6<br><br>Age at diagnosis & at study mean 8.3yr<br><br>Follow up minimum 2yrs	Perthes disease<br><br>Health professionals involved: A single observer administered the PODCI via telephone. The surgical procedure was carried out by the same senior orthopaedic surgeon		Modified Elizabethtown Classification	*Clinical*<br><br>PODCI administered via telephone, a minimum of two years following the surgery
Vinayak 2023 [25]<br><br>Retrospective, single centre<br><br>India	2012-2019<br><br>n=35<br><br>n with AVN=35<br><br>M=28, F=7	Perthes disease<br><br>Health professionals involved: operation completed by 2 senior paediatric orthopaedic surgeons,	Hip ROM<br><br>Radiological imaging:<br>AP and frog leg:<br><br>Acetabular height and depth	Modified Elizabeth Town Classification	*Radiological*<br><br>Stulberg grading<br><br>*Clinical*<br><br>Harris Hip score

	Age at diagnosis mean 8.1yr<br><br>Mean age at surgery 9.7yr<br><br>Follow up mean 3yr	post operative evaluation completed by a single researcher	Lateral extrusion of femoral head<br><br>Medial joint space<br><br>Shelf width and height<br><br>Sharp's angle<br><br>CEA	The Herring Lateral Pillar Classification	
Xiao 2023 [26]<br><br>Retrospective, single centre<br><br>China	2007-2016<br><br>n=13 (13 hips)<br><br>n with AVN=13<br><br>M=11, F=2<br><br>Age at diagnosis median 8yr<br><br>Mean age at study 8.4yr	Perthes disease<br><br>Health professionals involved: 2 independent orthopaedic surgeons assessed lateral pillar pre surgery. Physical therapists guided rehabilitation	Limp<br><br>Limb length discrepancy<br><br>Hip ROM<br><br>Radiological imaging: AP	Lateral Pillar Classification	*Radiological*<br><br>Modified Stulberg Classification<br><br>*Clinical*<br><br>Oucher pain scale

	Follow up 5.8yr				
Afaque 2024 [27]<br><br>Prospective, single centre<br><br>India	2019–2022<br><br>n=50<br><br>n with AVN=50<br><br>M=21, F=29<br><br>Mean age 8.14yr<br><br>Follow up not specified	Perthes disease<br><br>Health professionals involved: does not specify	Limping	The Herring Lateral Pillar Classification<br><br>Catterall Classification	
Das 2024 [28]<br><br>Retrospective, single centre<br><br>India	2008–2018<br><br>n=75<br><br>n with AVN=80<br><br>M=63, F=17	Perthes disease<br><br>Health professionals involved: does not specify	Hip ROM<br><br>Trendelenburg<br><br>Limping	The Herring Lateral Pillar Classification<br><br>Catterall Classification	*Radiological*<br><br>Stulberg Classification<br><br>*Clinical*<br><br>Pain - VAS

	Mean age at diagnosis 7.6yr<br><br>Mean age at surgery 9.4yr<br><br>Follow up 2yr		Leg length discrepancy<br><br>Radiological imaging:<br>CEA<br>Sharp's angle<br>Joint Space<br>Acetabular covering percentage<br>Neck Shaft angle<br>Articulo-trochanteric distance<br>Epiphyseal quotient		
Hoseini-Zare 2024 [29]<br><br>Retrospective, single centre	2006–2020<br><br>n=50<br><br>n with AVN=50	Perthes disease<br><br>Health professionals involved: single experienced surgical team	Hip ROM – abduction measured with goniometer, considered normal in >45º	The Herring Lateral Pillar Classification	*Radiological*<br><br>Stulberg Classification<br><br>*Clinical*

Iran	M=38, F=12<br><br>Mean age at surgery 7.9yr<br><br>Mean follow up 3.1yr		Trendelenburg – positive if pelvic dipped within 30 seconds of standing on affected limb<br><br>Radiological: AP and Laudenstein lateral, assessed pre and at final follow up<br>Acetabular index<br>CCD angle<br>CEA<br>Epiphyseal index<br>Extrusion index<br>Articulotrochanteric distance	Waldenström Classification of Perthes Disease	Harris Hip Score<br><br>Pain – VAS

Masse 2024 [30]<br><br>Retrospective, multicentre<br><br>Italy	2015–2021<br><br>n=11<br><br>n hips=13<br><br>n with AVN=13<br><br>M=7, F=4<br><br>Mean age at surgery 11.4yr<br><br>Mean follow up 3.3yr	Perthes disease<br><br>Health professionals involved: orthopaedic surgeons paediatric not specified	Hip ROM<br><br>Radiological:<br>CEA<br>Extrusion index<br>Caput index<br>Superior (Shenton line breakage)	MRI<br><br>Radiographs	*Clinical*<br><br>Modified Harris Hip Score<br><br>Pain – VAS<br>EQ-5D-5L<br><br>Merle d'Aubigné-Postel score
Wadström 2024 [31]<br><br>Retrospective, multicentre	2015–2024<br><br>n=309<br><br>n with AVN=309	Perthes disease<br><br>Health professionals involved: does not specify	Hip ROM<br><br>Trendelenburg<br><br>Radiological:	The Herring Lateral Pillar Classification	

	M=238, F=71<br><br>Mean age at diagnosis 6yr<br><br>Mean follow up 2yr		Reimers index	Modified Elizabeth Town Classification	
Valencia 2025 [32]<br><br>Retrospective, single centre<br><br>United States	2018–2022<br><br>n=20<br><br>n with AVN=20<br><br>M=18, F=2<br><br>Mean age at diagnosis 7.8yr<br><br>Mean age at surgery 8.2yr	Perthes disease<br><br>Health professionals involved: paediatric orthopaedic surgeons		Waldenström Classification of Perthes Disease	*Clinical*<br>PROMIS (mobility, anxiety, depressive symptoms, fatigue pain interference, peer relationships, and anger)<br>5–7yr parent as proxy reporters, >8yr self-reported

	Mean follow up 17 months				

n: number; AVN: avascular necrosis; M: male; F: female; yr: years; mon: months; Perthes disease: Legg–Calve–Perthes disease; DDH: Developmental dysplasia of the hip; SCD: Sickle Cell disease, SCFE: Slipped capital femoral epiphysis; ROM: range of motion; cm: centimetres; mm: millimeters; WB: Weightbearing ; BMI: Body mass index; FABER: flexion-abduction-external rotation; FADIR: flexion-adduction-internal rotation; AP: anterior posterior x-ray; 3D CT; three dimensional; computed tomography; MRI: magnetic resonance imaging; CEA: Centre-edge angles; CCD: Centrum-collum-diaphysis angle; ACM angle: Acetabular depth angle; VAS: visual analogue scale; EQ-5D-Y: EquroQual-5 Dimension 5 questions; PODCI: Paediatric Outcome Data Collection Instrument; PROMIS: Patient-Reported Outcomes Measurement Information System; WOMAC: Western Ontario and McMaster Universities Osteoarthritis Index.

References

1. **Amer AR, Khanfour AA**. Arthrodiastasis for late onset Perthes' disease using a simple frame and limited soft tissue release: early results. *Acta Orthop Belg*. 2011;77(4):472-9.
2. **Moghadam MH, Moradi A, Omidi-Kashani F**. Clinical outcome of femoral osteotomy in patients with legg-calve'-perthes disease. *Arch Bone Joint Surg*. 2013;1(2):90-3.
3. **Yavuz U, Demir B, Yildirim T, Beng K, Karakas ES**. Salter innominate osteotomy in the treatment of late presentation Perthes disease. *Hip Int*. 2014;24(1):39-43. doi:https://dx.doi.org/10.5301/hipint.5000086
4. **Camurcu IY, Yildirim T, Buyuk AF, Gursu SS, Bursali A, Sahin V**. Tonnis triple pelvic osteotomy for Legg-Calve-Perthes disease. *Int Orthop*. 2015;39(3):485-90. doi:https://dx.doi.org/10.1007/s00264-014-2585-6
5. **Elzohairy M, Elzohairy MM**. Short follow-up evaluation of proximal femoral varus osteotomy for treatment of Legg-Calvé-Perthes disease. *J Orthop Traumatol*. 2016;17(4):345-351. doi:10.1007/s10195-016-0412-0
6. **Bhuyan BK**. Early outcomes of one-stage combined osteotomy in Legg-Calve'-Perthes disease. *Indian J Orthop*. 2016;50(2):183-94. doi:https://dx.doi.org/10.4103/0019-5413.177581
7. **Aguado-Maestro I, Abril JC, Banuelos Diaz A, Garcia Alonso M**. Hip arthrodiastasis in Legg-Calve-Perthes disease. *Rev Esp Cir Ortop Traumatol*. 2016;60(4):243-50. doi:https://dx.doi.org/10.1016/j.recot.2016.03.002
8. **Keun-Sang K, Sung Il W, Ju-Hyung L, et al**. Effect of greater trochanteric epiphysiodesis after femoral varus osteotomy for lateral pillar classification B and B/C border Legg-Calvé-Perthes disease: A retrospective observational study. *Medicine*. 2017;96(31):1-6. doi:10.1097/MD.0000000000007723

9. **Joshi N, Mohapatra S, Goyal M, Goyal S, Kumar R, Saini M**. Short Term Outcome of Varus Derotation Osteotomy in Late Presenting Perthes Disease. *Indian J Orthop*. 2018;52(2):133-139. doi:10.4103/ortho.IJOrtho_196_16

10. **Kanatli U, Ayanoglu T, Ozer M, Ataoglu MB, Cetinkaya M**. Hip arthroscopy for Legg-Calve-Perthes disease in paediatric population. *Acta Orthop Traumatol Turc*. 2019;53(3):203-208. doi:https://dx.doi.org/10.1016/j.aott.2019.03.005

11. **Leo DG, Murphy R, Gambling T, Long A, Jones H, Perry DC**. Perspectives on the Social, Physical, and Emotional Impact of Living With Perthes' Disease in Children and Their Family: A Mixed Methods Study. *Glob Pediatr Health*. 2019;6doi:10.1177/2333794X19835235

12. **Matsumoto H, Hyman JE, Shah HH, et al**. Validation of Pediatric Self-Report Patient-Reported Outcomes Measurement Information System (PROMIS) Measures in Different Stages of Legg-Calvé-Perthes Disease. *J Pediatr Orthop*. 2020;40(5):235-240. doi:10.1097/BPO.0000000000001423

13. **Haskel JD, Feder OI, Mijares J, Castaneda P**. Isolated Trochanteric Descent and Greater Trochanteric Apophyseodesis Are Not Effective in the Treatment of Post-Perthes Deformity. *Clin Orthop Relat Res*. 2020;478(1):169-175. doi:https://dx.doi.org/10.1097/CORR.0000000000000990

14. **El-Harbiti A, Hailer YD**. Range of abduction in patients with Legg-Calve-Perthes disease - a nationwide register-based cohort study. *BMC Musculoskelet Disord*. 2020;21(1):718. doi:https://dx.doi.org/10.1186/s12891-020-03705-4

15. **Laine JC, Novotny SA, Tis JE, et al**. Demographics and Clinical Presentation of Early-Stage Legg-Calvé-Perthes Disease: A Prospective, Multicenter, International Study. *J Am Acad Orthop Surg*. 2021;29(2):e85-e91. doi:10.5435/JAAOS-D-19-00379

16. **Dang-Huy D, McGuire MF, Chan-hee J, Kim HKW, Do D-H, Jo C-H**. Weightbearing and Activity Restriction Treatments and Quality of Life in Patients with Perthes Disease. *Clin Orthop Relat Res*. 2021;479(6):1360-1370. doi:10.1097/CORR.0000000000001608

17. **Do D-H, Valencia AA, Jo C-h, Kim HKWMS, Kim HKW**. Moderate Weightbearing Restrictions Are Associated with Worse Depressive Symptoms and Anxiety in Children Aged 5 to 7 Years with Perthes Disease. *Clin Orthop Relat Res*. 2022;480(3):587-599. doi:10.1097/CORR.0000000000002010

18. **Ghaznavi A, Motaghi P, Ghaderi MT, Sileymani N, Saberi S, Mohammadpour M**. Short-term radiographic outcome of varus osteotomy in the treatment of Legg-Calve-Perthes disease: A retrospective case series study. *Curr Orthop Pract*. 2022;33(3):277-281. doi:https://dx.doi.org/10.1097/BCO.0000000000001106

19. **Perry DC, Arch B, Appelbe D, et al**. The British Orthopaedic Surgery Surveillance study: Perthes' disease: the epidemiology and two-year outcomes from a prospective cohort in Great Britain. *Bone Joint J*. 2022;104-B(4):510-518. doi:https://dx.doi.org/10.1302/0301-620X.104B4.BJJ-2021-1708.R1

20. **Al-Osaimi MN, Alsubaihi AA, Basaqr AA**. Management of Patients With Legg-Calve-Perthes Disease at a Single Center in Jeddah, Saudi Arabia. *Cureus*. 2022;14(6):e26262. doi:https://dx.doi.org/10.7759/cureus.26262

21. **Arsalan M, Khan M, Shahid M, Qamar Z, Akhlaq S, Ullah A**. Principles of Treating the Sequelae of Perthes Disease. *Pak J Med Health Sci*. 2023;17(5):290-293. doi:https://dx.doi.org/10.53350/pjmhs2023175290

22. **Eidelman M, Kotlarsky P**. Does transepiphyseal drilling and closure of the greater trochanter in early Legg-Calve-Perthes disease improve natural history? *Musculoskelet Surg*. 2023;107(3):279-285. doi:https://dx.doi.org/10.1007/s12306-022-00750-1

23. **Gumin Tayeng A, Sengupta A, Chaterjee P**. Clinical Outcomes of Open-Wedge Varus Derotation Osteotomy in Legg-Calve-Perthes Disease Among 6-12-Year-Old Children. *Cureus*. 2023;15(6):e41144. doi:https://dx.doi.org/10.7759/cureus.41144

24. **Lal JV, Tontanahal S, Francis J, Philip KM, Lj R, Kurian BT**. Functional Outcome of Varus Derotation Osteotomy in Legg-Calve-Perthes Disease: Can It Be Justified in Late-Presenting Disease? *Cureus*. 2023;15(12):e49788. doi:https://dx.doi.org/10.7759/cureus.49788

25. **Vinayak U, Gundawar C, Shyam A, et al**. Labral Support Shelf Acetabuloplasty for Late Presenting Perthes Disease: Outcomes in Indian Patients. *Indian J Orthop*. 2023;57(11):1785-1792. doi:https://dx.doi.org/10.1007/s43465-023-00978-4

26. **Xiao YB, Du W, Wu PF, Qing LM, Yu F, Tang JY**. Pedicled iliac bone flap grafting in the treatment of late presentation Legg-Calve-Perthes disease. *Front Surg*. 2023;10:926109. doi:https://dx.doi.org/10.3389/fsurg.2023.926109

27. **Afaque SF, Verma V, Agrawal U, Chand S, Singh V, Singh A**. The Effect of Vitamin D Deficiency as a Risk Factor of Early Fragmentation in Legg-Calve-Perthes Disease: A Prospective Study. *Curēus (Palo Alto, CA)*. 2024;16(3):e57274-e57274. doi:10.7759/cureus.57274

28. **Bihari Das P, Ranjan Mishra N, Krishnan Ganesh A, Mohanty S, Ranjan Dash R, Prasad Das S**. Comparative study between lateral shelf acetabuloplasty and combined procedure of lateral shelf acetabuloplasty with trochanteric epiphysiodesis in cases of Legg-Calves-Perthes disease — a retrospective study. *Ortop, Travmat Prot*. 2024;(4):48-57. doi:10.15674/0030-59872024448-57

29. **Hoseini-Zare N, Mirghaderi P, Ilharreborde B, et al**. Proximal femoral varus osteotomy for Legg–Calvé–Perthes disease: Do age and lateral pillar classifications influence short-to-mid-term clinical and radiological outcomes? *Orthop Traumatol Surg Res*. 2024:103909. doi:10.1016/j.otsr.2024.103909

30. **Massè A, Giachino M, Audisio A, et al**. Ganz femoral head reduction associated with coverage and containment procedures improve radiological and functional outcomes in Perthes' disease. *Bone Joint J*. 2024;106-B(5 Supple B):40-46. doi:10.1302/0301-620X.106B5.BJJ-2023-0853.R1

31. **Wadström MG, Hailer NP, Hailer YD**. Demographics and risk for containment surgery in patients with unilateral Legg-Calvé-Perthes disease: a national population-based cohort study of 309 patients from the Swedish Pediatric Orthopedic Quality Register. *Acta Orthop*. 2024;95:333-339. doi:10.2340/17453674.2024.40907

32. **Valencia AA, Do D-H, Jo C-H, Kim HKW**. Longitudinal Improvement of Quality of Life in Children With Legg-Calve-Perthes Disease Treated With Proximal Femoral Varus Osteotomy. *J Pediatr Orthop*. 2025. doi:10.1097/BPO.0000000000002896
