# Supplementary material for: Variation in assessment, diagnosis and outcome measurement in Perthes disease: a scoping review
Source: J Child Orthop. 2026 Mar 24;20(3):247–55. doi: 10.1177/18632521261432861 (PMC13017626; doi:10.1177/18632521261432861)
Supplement: sj-pdf-3-cho-10.1177_18632521261432861 – Supplemental material for Variation in assessment, diagnosis and outcome measurement in Perthes disease: a scoping review [file sj-pdf-3-cho-10.1177_18632521261432861.pdf]

Supplemental Table 2: Linking Outcome Measures to the Core Outcome Set for Perthes Disease, Psychometric Properties and Original Development

Outcome measure	n (%) studies using outcome measure	Psychometrically valid for children	Psychometrically valid for Perthes disease	Originally developed for children	Core outcome domains
**Radiological outcome measures**					
Stulberg Hip Classification [1]	9 (28.1)	✘	✓ [2]	✓	Acetabular coverage and hip congruence<br>Femoral head shape<br>Evidence of arthritic changes
Modified Stulberg Hip Classification [2]	3 (9.4)	✘	✓ [2, 3]	✓	Acetabular coverage and hip congruence<br>Femoral head shape
Mose's Method [4]	1 (3.1)	✘	✘	✘	Acetabular coverage and hip congruence<br>Femoral head shape
Ratliff criteria [5]	1 (3.1)	✘	✘	✓ [5]	Pain<br>ADL<br>Hip mobility<br>Evidence of arthritic changes
Catterall post operative classification [6, 7]	1 (3.1)	✘	✘	✓	Acetabular coverage and hip congruence<br>Femoral head shape
**Clinical outcome measures**					
Harris Hip Score [8]	7 (21.9)	✘	✘	✘ [9]	Pain<br>ADL<br>Hip mobility

Pain – VAS [10]	5 (15.6)	✓ [11]	✗	✗	Pain
PROMIS^^ [12] (child/adolescent and parent as proxy reporters)	3 (9.4)	✓	✓ [13]	✗	Pain<br>ADL<br>Psychological impact<br>QOL
Modified Harris Hip Score [14, 15]	2 (6.2)	✗	✗	✗	Pain<br>ADL
Iowa Hip Score [16]	2 (6.2)	✗	✗	✗ [16]	Pain<br>ADL<br>Hip mobility
PedsQL 4.0 [17]	1 (3.1)	✓ [18]	✗	✓	QOL
WOMAC Score [19]	1 (3.1)	✗	✗	✗	Pain<br>ADL<br>Hip mobility<br>QOL
EQ-5D-5L [20] (child/adolescent and parent as proxy reporters)	2 (6.2)	✓ [21]	✓	✓ [22]	Pain<br>ADL<br>Psychological impact<br>QOL
PODCI [23]	1 (3.1)	✓ [24]	✓ [24]	✓ [23]	Pain<br>ADL<br>Psychological impact<br>Sport participation
Bespoke questionnaire	1 (3.1)	✗	✗	✓	Pain<br>ADL<br>Psychological impact<br>QOL
Parental interview	1 (3.1)	✗	✗	✗	Pain<br>ADL

					Psychological impact<br>QOL<br>Family life
Wong-Baker FACES scale** [25]	1 (3.1)	✓ [25-27]	✗	✓	Pain
Oucher Pain Scale	1 (3.1)	✓ [28]	✗	✓	Pain
Numeric Pain scale [29]	1 (3.1)	✓ [29]	✗	✗	Pain
Missing days from school	1 (3.1)	✗	✗	✓	School/preschool attendance
Merle d’Aubigné Postel Score [30]	1 (3.1)	✗	✗	✗	Pain<br>ADL

VAS: Visual analogue scale; PROMIS: Patient reported outcome measurement information system; PedsQL4.0: Pediatric Quality of Life Inventory; WOMAC: Western Ontario and McMaster Universities Osteoarthritis Index; EQ-5D-5L: EquroQual-5 Dimension 5 questions; PODCI: Pediatric Outcomes Data Collection Instrument; ^^: PROMIS mobility, paediatric measures, and parent proxy measures; **: Wong Baker FACES Pain Rating Scale; ✓: Yes; ✗: No.

References

1. **Stulberg SD**. The natural history of Legg-Calve-Perthes disease. *J Bone Joint Surg Am*. 1981;63:1095-1108.

2. **Wiig O, Terjesen T, Svenningsen S**. Inter-observer reliability of the Stulberg classification in the assessment of Perthes disease. *J Child Orthop*. 2007;1(2):101-105. doi:10.1007/s11832-007-0020-x

3. **Huhnstock S, Wiig O, Merckoll E, Svenningsen S, Terjesen T**. The modified Stulberg classification is a strong predictor of the radiological outcome 20 years after the diagnosis of Perthes' disease. *J Bone Joint Surg Br*. 2021;103-B(12):1815-1820. doi:10.1302/0301-620X.103B12.BJJ-2021-0515.R1

4. **Mose K**. Methods of measuring in Legg-Calve-Perthes disease with special regard to the prognosis. *Clin Orthop Relat Res*. 1980;150(150):103-109.

5. **Ratliff AH**. Pseudocoxalgia; a study of late results in the adult. *J Bone Joint Surg Br*. 1956;38(2):498-512. doi:10.1302/0301-620x.38b2.498

6. **Catterall A**. The Natural History of Perthes Disease. *J Bone Joint Surg Br*. 1971;53:37-53.

7. **Than P, Halmai V, Shaikh S, Kránicz J, Bellyei Á**. Long-Term Results of Derotational Femoral Varus Osteotomy in Legg-Calvé-Perthes Disease: 26-Year Follow-Up. *Orthopedics (Thorofare, NJ)*. 2003;26(5):487-491. doi:10.3928/0147-7447-20030501-13

8. **Christensen CP, Althausen PL, Mittleman MA, Lee Ja, McCarthy JC**. The nonarthritic hip score: Reliable and validated. *Clin Orthop Relat Res*. 2003;406(406):75-83. doi:10.1097/00003086-200301000-00013

9. **Harris WH**. Traumatic Arthritis of the Hip after Dislocation and Acetabular Fractures: Treatment by Mold Arthroplasty: AN END-RESULT STUDY USING A NEW METHOD OF RESULT EVALUATION. *J Bone Joint Surg Am*. 1969;51(4):737-755. doi:10.2106/00004623-196951040-00012

10. **Powell CV, Kelly A-M, Williams A**. Determining the minimum clinically significant difference in visual analog pain score for children. *Ann Emerg Med*. 2001;37(1):28-31. doi:10.1067/mem.2001.111517

11. **Goodenough B, Addicoat L, Champion GD, et al**. Pain in 4- to 6-Year-Old Children Receiving Intramuscular Injections: A Comparison of the Faces Pain Scale with

Other Self-Report and Behavioral Measures. *Clin J Pain*. 1997;13(1):60-73. doi:10.1097/00002508-199703000-00009

12. **Ader DN**. Developing the Patient-Reported Outcomes Measurement Information System (PROMIS). *Med Care*. 2007;45:S1-2.

13. **Matsumoto H, Hyman JE, Shah HH, et al**. Validation of Pediatric Self-Report Patient-Reported Outcomes Measurement Information System (PROMIS) Measures in Different Stages of Legg-Calvé-Perthes Disease. *J Rediatr Orthop*. 2020;40(5):235-240. doi:10.1097/BPO.0000000000001423

14. **Kumar P, Sen R, Aggarwal S, Agarwal S, Rajnish RK**. Reliability of Modified Harris Hip Score as a tool for outcome evaluation of Total Hip Replacements in Indian population. *J Clin Orthop Trauma*. 2019;10(1):128-130. doi:10.1016/j.jcot.2017.11.019

15. **Stasi S, Papathanasiou G, Diochnou A, Polikreti B, Chalimourdas A, Macheras GA**. Modified Harris Hip Score as patient-reported outcome measure in osteoarthritic patients: psychometric properties of the Greek version. *Hip Int*. 2021;31(4):516-525. doi:10.1177/1120700020901682

16. **Larson CB**. Rating scale for hip disabilities. *Clin Orthop Relat Res*. 1963;31(1):85-93. doi:10.1097/00003086-196300310-00011

17. **Varni JW, Seid M, Rode CA**. The PedsQL™: Measurement Model for the Pediatric Quality of Life Inventory. *Med Care*. 1999;37(2):126-139. doi:10.1097/00005650-199902000-00003

18. **Varni JW, Seid M, Kurtin PS**. PedsQL™ 4.0: Reliability and Validity of the Pediatric Quality of Life Inventory™ Version 4.0 Generic Core Scales in Healthy and Patient Populations. *Med Care*. 2001;39(8):800-812. doi:10.1097/00005650-200108000-00006

19. **Roos M, S. KL, M. LE**. WOMAC Osteoarthritis Index: Reliability, validity, and responsiveness in patients with arthroscopically assessed osteoarthritis. *Scand J Rheumatol*. 1999;28(4):210-215. doi:10.1080/03009749950155562

20. **Stolk E, Ludwig K, Rand K, van Hout B, Ramos-Goñi JM**. Overview, Update, and Lessons Learned From the International EQ-5D-5L Valuation Work: Version 2 of the EQ-5D-5L Valuation Protocol. *Value in Health*. 2019;22(1):23-30. doi:10.1016/j.jval.2018.05.010

21. **Bilbao A, García-Pérez L, Arenaza JC, et al**. Psychometric properties of the EQ-5D-5L in patients with hip or knee osteoarthritis: reliability, validity and responsiveness. *Quality of life research*. 2018;27(11):2897-2908. doi:10.1007/s11136-018-1929-x

22. **EuroQol Research Foundation**. EQ-5D-5L User Guide, 2025. Available from: https://euroqol.org/information-and-support/euroqol-instruments/eq-5d-5l/

23. **Scott KS, Barbosa GO, Pan J, Heathcock JC**. Using the PODCI to Measure Motor Function and Parent Expectations in Children With Cerebral Palsy. *Phys Ther*. 2021;101(12)doi:10.1093/ptj/pzab215

24. **Daltroy LH, Liang MH, Fossel AH, Goldberg MJ**. The POSNA Pediatric Musculoskeletal Functional Health Questionnaire: Report on Reliability, Validity, and Sensitivity to Change. *J Pediatr Orthop*. 1998;18(5):561-571. doi:10.1097/00004694-199809000-00001

25. **Garra G, Singer AJ, Domingo A, Thode HC**. The Wong-Baker Pain FACES Scale Measures Pain, Not Fear. *PediatrEemerg Care*. 2013;29(1):17-20. doi:10.1097/PEC.0b013e31827b2299

26. **Bieri D, Reeve RA, Champion GD, Addicoat L, Ziegler JB**. The faces pain scale for the self-assessment of the severity of pain experienced by children: Development, initial validation, and preliminary investigation for ratio scale properties. *Pain*. 1990;41(2):139-150. doi:10.1016/0304-3959(90)90018-9

27. **Chambers CT, Giesbrecht K, Craig KD, Bennett SM, Huntsman E**. A comparison of faces scales for the measurement of pediatric pain: children's and parents' ratings. *Pain*. 1999;83(1):25-35. doi:10.1016/S0304-3959(99)00086-X

28. **Beyer JE, Turner SB, Jones L, Young L, Onikul R, Bohaty B**. The alternate forms reliability of the Oucher pain scale. *Pain Manag Nurs*. Mar 2005;6(1):10-7. doi:10.1016/j.pmn.2004.11.001

29. **Tsze DS, von Baeyer CL, Pahalyants V, Dayan PS**. Validity and Reliability of the Verbal Numerical Rating Scale for Children Aged 4 to 17 Years With Acute Pain. *Ann Emerg Med*. Jun 2018;71(6):691-702.e3. doi:10.1016/j.annemergmed.2017.09.009

30. **Merle D'Aubigne R**. Functional results of hip arthroplasty with acrylic prosthesis. *J Bone Joint Surg Am*. 1954;36:451-475.
